# Second-Line Treatment Strategies for Right-Sided, RAS/RAF Wild-Type Colorectal Cancer

**DOI:** 10.1001/jamanetworkopen.2025.15087

**Published:** 2025-06-12

**Authors:** Nishwant Swami, Wei-Ting Hwang, Ronac Mamtani, Mark H. O’Hara, William J. Chapin

**Affiliations:** 1 Department of Internal Medicine, Hospital of the University of Pennsylvania, Philadelphia; 2 Department of Biostatistics, Epidemiology and Informatics, University of Pennsylvania Perelman School of Medicine, Philadelphia; 3 Division of Hematology and Oncology, Department of Medicine, University of Pennsylvania Perelman School of Medicine, Philadelphia

## Abstract

This comparative effectiveness study investigates anti–vascular endothelial growth factor vs anti–epidermal growth factor receptor therapy in second-line treatment among patients with RAS/RAF wild-type, right-sided cancer.

## Introduction

For patients with metastatic colorectal cancer (mCRC) that is wild type for *KRAS, NRAS*, and *BRAF* (RAS/RAF), treatment includes fluoropyrimidine-based chemotherapy with anti–vascular endothelial growth factor (VEGF) or anti–epidermal growth factor receptor (EGFR) targeted therapy. The PARADIGM trial confirmed that primary tumor sidedness was a predictive biomarker for patients with RAS/RAF wild-type disease and showed no overall survival (OS) benefit from first-line chemotherapy plus anti-EGFR therapy compared with chemotherapy plus anti-VEGF therapy for patients with right-sided tumors.^1,2^ However, to our knowledge, there are no prospective data to guide second-line treatment in this population.^3^ Despite limited evidence, national guidelines recommend anti-EGFR therapy for patients with RAS/RAF wild-type, right-sided cancer who did not receive it previously.^4^ To better inform practice, we compared the effectiveness of second-line chemotherapy plus anti-EGFR vs chemotherapy plus anti-VEGF therapy for patients with RAS/RAF wild-type, right-sided mCRC who received first-line chemotherapy plus anti-VEGF.

## Methods

This comparative effectiveness study was exempted from review by the institutional review board at the University of Pennsylvania by meeting 45 CFR §46.104, category 4. It is reported following the ISPOR reporting guideline. We used the nationwide, longitudinal Flatiron Health electronic health record–derived database, comprising deidentified, patient-level structured and unstructured data curated via technology-enabled abstraction from approximately 280 cancer clinics (approximately 800 care sites), most being community oncology settings.^5,6^ Data are deidentified and subject to obligations to prevent reidentification and protect patient confidentiality. Patients 18 years or older with RAS/RAF wild-type, right-sided mCRC who received first-line therapy of chemotherapy plus anti-VEGF and second-line therapy with anti-VEGF therapy (bevacizumab or its biosimilars, ziv-aflibercept, or ramucirumab) or anti-EGFR therapy (cetuximab or panitumumab) between January 2013 and May 2024 were eligible (eMethods in Supplement 1). Multiple imputation with chained equations (MICE) imputed missing values for tumor sidedness, RAS/RAF status, and propensity score model covariates (age, gender, year of diagnosis, synchronous or metachronous metastases, mismatch repair and microsatellite instability status, performance status, carcinoembryonic antigen level, and duration of first-line therapy) (eMethods and eTable in Supplement 1). After MICE, we used Cox proportional hazards modeling with stabilized inverse probability of treatment weighting (IPTW) to assess the adjusted association of anti-EGFR vs anti-VEGF–containing treatment with OS using Stata/SE statistical software version 18.5 (StataCorp) (eMethods, eFigure 1, and eFigure 2 in Supplement 1). A 2-sided *P* < .05 was considered statistically significant.

## Results

A total of 4444 patients received first-line therapy plus anti-VEGF therapy and second-line anti-VEGF or anti-EGFR therapy with chemotherapy; data were missing for 709 patients (16.0%) on tumor sidedness and 993 patients (22.3%) on RAS/RAF status. Across 25 imputations, a mean of 444 patients (median [IQR] age, 65 [56-72] years; 175 women [39.4%]) met inclusion criteria; 269 patients received anti-VEGF therapy, and 175 patients received anti-EGFR therapy (Table). After IPTW, baseline characteristics were well balanced between treatment groups. Patients who received chemotherapy plus anti-EGFR therapy did not have a significantly increased hazard of death compared with patients who received chemotherapy plus anti-VEGF therapy (hazard ratio, 1.24; 95% CI, 0.96-1.61; *P* = .10) (Figure). The estimated median survival from the IPTW-adjusted Cox proportional hazards model was 15.3 months (95% CI, 12.5-17.1 months) among patients with chemotherapy plus anti-VEGF therapy and 12.0 months (95% CI, 9.8-15.3 months) among those receiving chemotherapy plus anti-EGFR therapy.

**Table.** Study Population Characteristics

Characteristic	Patients, No. (%)		
	Total cohort (N = 444)^a^	Anti-VEGF therapy (n = 269)^a^	Anti-EGFR therapy (n = 175)^a^
Age, median (IQR), y	65 (56-72)	65 (56-72)	65 (56-71)
Gender			
Women	175 (39.4)	105 (39.0)	70 (40.0)
Men	269 (60.6)	164 (61.0)	105 (60.0)
ECOG performance status category			
0-1	390 (87.8)	243 (90.5)	147 (84.1)
≥2	54 (12.2)	26 (9.5)	28 (15.9)
MMR or MSI status			
MMR proficient, MSS, or both	422 (95.1)	256 (95.4)	166 (94.6)
MMR deficient, MSI high, or both	22 (4.9)	12 (4.6)	10 (5.4)
CEA within 30 d of index date, median (IQR)	23 (6.3-89.1)	21.8 (6.1-80.4)	25.4 (6.5-111.9)
Duration of first-line treatment, median (IQR), d	309 (181-510)	309 (182-510)	308 (176-517)
Timing of metastasis			
Synchronous^b^	330 (74.3)	206 (76.8)	124 (70.5)
Metachronous	114 (25.7)	62 (23.2)	52 (29.5)

Abbreviations: CEA, carcinoembryonic antigen; ECOG, Eastern Cooperative Oncology Group; EGFR, epidermal growth factor receptor; MMR, mismatch repair; MSI, microsatellite instability; MSS, microsatellite stable; VEGF, vascular endothelial growth factor.

^a^ Study population is the primary cohort after multiple imputation. Anti-VEGF and anti-EGFR therapy were received with second-line chemotherapy. Provided medians and percentages were calculated for the primary analysis cohort after multiple imputation with chained equations performed across 25 imputations for all presented covariates. Sample sizes reflect means across the 25 imputations.

^b^ Metastatic disease is defined as synchronous if the date of metastasis is within 90 days of the initial cancer diagnosis and as metachronous if the date of metastasis is more than 90 days after the initial diagnosis.

**Figure.**
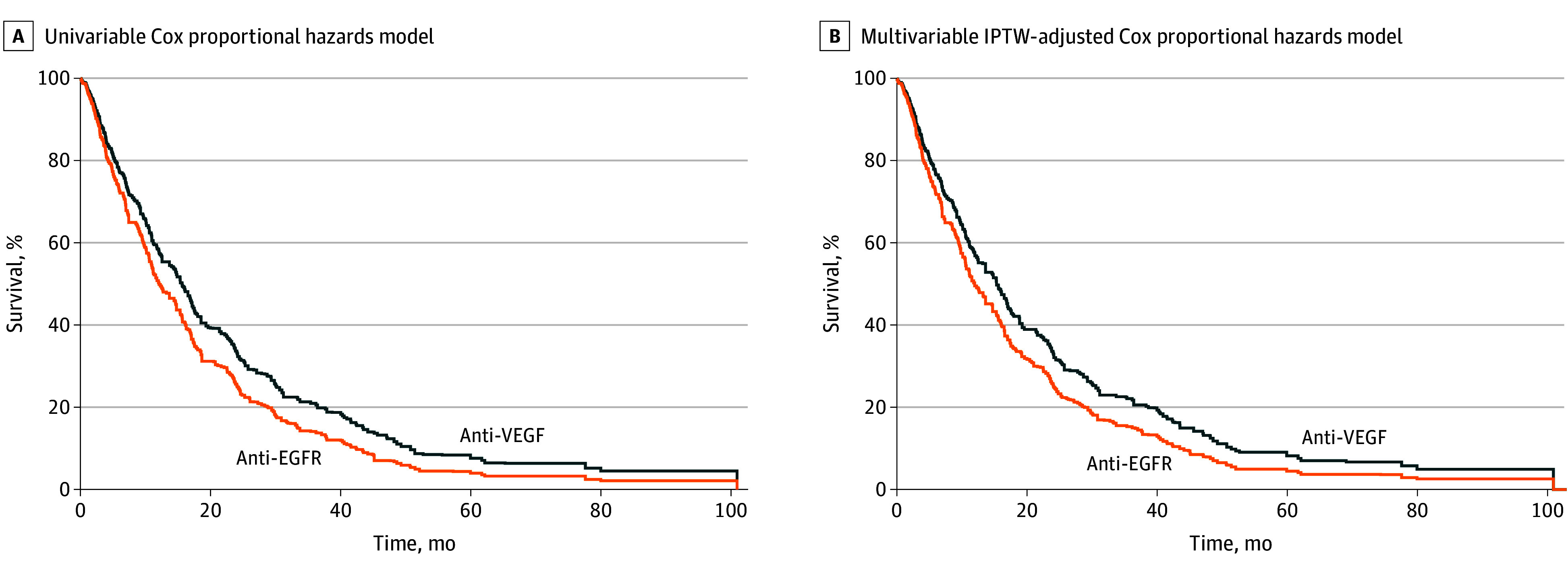
Estimated Survival Functions Estimated survival functions were generated independently for each of 25 imputations, with the mean subsequently calculated at each time interval to generate the estimated survival function after multiple imputation. Because the plot represents means of estimated survival functions and not directly observed data, the number at risk at each time are not provided. Estimated median survival was 15.6 months (95% CI, 12.5-17.1 months) in unadjusted and 15.3 months (95% CI, 12.5-17.1 months) in adjusted analysis for patients treated with anti–vascular endothelial growth factor (VEGF) therapy and 12.0 months (95% CI, 9.8-15.3 months) in unadjusted and 12.1 months (95% CI, 10.5-15.3 months) in adjusted analysis for patients treated with anti–epidermal growth factor receptor (EGFR) therapy. IPTW indicates stabilized inverse probability of treatment weights.

## Discussion

Among patients with RAS/RAF wild-type, right-sided mCRC who received first-line chemotherapy plus anti-VEGF therapy, this comparative effectiveness research’s findings provide some evidence for continuing anti-VEGF therapy in the second line, although the result was not statistically significant. Our findings corroborate outcomes in first-line therapy from the PARADIGM trial, where nonsignificantly worse OS was observed in patients with right-sided disease receiving anti-EGFR compared with anti-VEGF therapy (hazard ratio, 1.09; 95% CI, 0.79-1.51).^1^ Limitations include possible unmeasured confounding and limited power given the relatively small proportion of mCRC that is right sided and RAS/RAF wild type. Future studies should explore better predictive biomarkers for anti-EGFR vs anti-VEGF treatment in this population to identify patients most likely to benefit from anti-EGFR therapy at any time during their disease course.

## Notes

Supplement 1.
eMethods.
**eTable.** *ICD-9* and *ICD-10* diagnostic codes used to define tumor-sidedness **eFigure 1.** Cohort Selection of Patients **eFigure 2.** Covariate balance
eReferences.

Supplement 2.
Data Sharing Statement
