# Supplementary material for: Second-Line Treatment Strategies for Right-Sided, RAS/RAF Wild-Type Colorectal Cancer
Source: JAMA Netw Open. 2025 Jun 12;8(6):e2515087. doi: 10.1001/jamanetworkopen.2025.15087 (PMC12163663; doi:10.1001/jamanetworkopen.2025.15087)
Supplement: Supplement 1. — eMethods. eTable. ICD-9 and ICD-10 diagnostic codes used to define tumor-sidedness eFigure 1. Cohort Selection of Patients eFigure 2. Covariate balance eReferences. [file jamanetwopen-e2515087-s001.pdf]

# Supplemental Online Content

Swami N, Hwang W, Mamtani R, O'Hara MH, Chapin WJ. Second-line treatment strategies for right-sided, RAS/RAF wild-type colorectal cancer*.* *JAMA Netw Open*. 2025;8(6):e2515087. doi:10.1001/jamanetworkopen.2025.15087

**eMethods.**
**eTable.** *ICD-9* and *ICD-10* diagnostic codes used to define tumor-sidedness
**eFigure 1.** Cohort Selection of Patients
**eFigure 2.** Covariate balance
**eReferences.**

This supplemental material has been provided by the authors to give readers additional information about their work.

© 2025 Swami N et al. *JAMA Network Open*.

# 1. eMethods

## 1.1. Detailed inclusion and exclusion criteria:

Inclusion criteria: Patients ≥ 18 years old with *KRAS, NRAS, and BRAF* (RAS/RAF) wild type, right-sided metastatic colorectal cancer who received first-line therapy consisting of chemotherapy + anti-VEGF therapy and subsequently received their first administration (index date) of second-line chemotherapy with either anti-VEGF therapy or anti-EGFR therapy between January 2013 – May 2024 were eligible.

RAS/RAF mutation status was a binary variable defined by *KRAS, NRAS,* and *BRAF* mutation status on tumor tissue closest to the index date, but within the window from any time prior to index date to 60 days following index date. Observations were categorized as RAS/RAF wild-type if wild-type for all three genes and as RAS/RAF altered if an alteration was present in any of the three genes, even if information was unavailable for one or both of the other genes. However, if no RAS/RAF alterations were present and there was missing information for at least one of the three genes, then the observation was coded as missing.

Tumor sidedness was a binary variable defined by presence of ICD9 or ICD10 codes specific for tumor sidedness, prior to and including the index date. This approach was modeled off that validated by Luhn et al. with several key differences.[1] Rather than choosing only diagnosis codes for colorectal cancer closest to the cancer diagnosis date as was performed in the validation study, we included only colorectal cancer codes that indicated a specific side of the colon (**eTable 1**) that were present prior to, or including the index date. Amongst these codes, the code closest to the index date was chosen for this determination. The second difference was that we did not include diagnosis codes for the anus or anal canal as sidedness-specific codes for our patients with metastatic colorectal cancer. From the ascending colon the splenic flexure (including all of the transverse colon; excluding splenic flexure) was considered right-sided while from the splenic flexure to rectum was considered left-sided. The specific diagnostic codes to determine sidedness are included in **eTable 1**. Only right-sided tumors were included in the study population.

First line chemotherapy regimens included in the study consisted of doublet (5-FU + oxaliplatin (FOLFOX), 5-FU + irinotecan (FOLFIRI), capecitabine + oxaliplatin (CAPEOX), capecitabine + irinotecan (CAPIRI)) or triplet chemotherapy regimens (5-FU, irinotecan, oxaliplatin) combined with anti-VEGF treatment (bevacizumab/biosimilars; ziv-aflibercept; ramucirumab). Second line chemotherapy regimens included in the study consisted of only doublet chemotherapy backbones (FOLFOX, FOLFIRI, CAPEOX, CAPIRI) combined with either anti-VEGF (bevacizumab/biosimilars; ziv-aflibercept; ramucirumab) or anti-EGFR therapy (cetuximab or panitumumab).

Exclusion criteria: Patients were excluded if death or censoring occurred on the index date or prior (see 1.3 for detailed definition of outcome).

## 1.2. Detailed definitions of covariates:

Pre-specified covariates considered to be potential confounders included age, gender, year of metastatic diagnosis, baseline carcinoembryonic antigen (CEA), mismatch repair deficiency or microsatellite instability status (MMR/MSI status), synchronous versus metachronous metastases, Eastern Cooperative Oncology Group (ECOG) performance status, and duration of first line treatment.

Age: Defined as the date of first administration of 2$^{nd}$ line systemic treatment minus birth-year. Coded as a continuous variable.

Gender: Self-reported by patients and recorded as a binary variable (man or woman).

Year of metastatic diagnosis: Binary variable defined by calendar year of diagnosis of metastatic colorectal cancer. Categories included < 2018 and >/= 2018.

© 2025 Swami N et al. *JAMA Network Open*.

ECOG performance status: Binary variable defined as ECOG status recorded within 30 days prior to 7 days after index date. Categories include ECOG 0 – 1 and ECOG >/= 2.

Mismatch repair (MMR)/microsatellite instability (MSI) Status: Binary variable defined by MMR/MSI status closest to the index date and occurring within the window of any time prior to index date to within 60 days following the index date. An observation was categorized as MMR deficient or MSI high (MMRd/MSI-H) if either mismatch repair deficiency or microsatellite instability-high status were present. Otherwise, the observation was coded as MMR proficient or microsatellite instability – low status (MMRp/MSS) if not missing.

Baseline CEA: Continuous variable defined by CEA measurement within 30 days prior to and including the index date. CEA is presented in units of micrograms per L.

Synchronous or Metachronous metastatic disease: Binary variable defined by the time from colon or rectal cancer diagnosis to the time of diagnosis of metastatic disease. Less than 90 days was defined as synchronous while greater than or equal to 90 days was defined as metachronous.

## 1.3. Detailed definition of outcome:

Overall survival: Time from first treatment administration (index-date) to death, with censoring for last confirmed activity including in-person visit or confirmation of treatment administration.

## 1.4. Statistical analysis:

### 1.4.1. Sample size determination

As this study was retrospective and used and existing dataset, sample size was determined by the number of patients meeting eligibility criteria.

### 1.4.2. Multiple imputation with chained equations

Missing values from pre-specified covariates, RAS/RAF status, and tumor sidedness were assumed to be missing at random (MAR) and multiple imputation with chained equations with 25 imputations was used to minimize bias related to missing cohort-defining variables and missing covariates. Following multiple imputation of missing covariates, RAS/RAF status, and tumor sidedness, multivariable logistic regression model with prespecified covariates based on clinical knowledge (eMethods 1.2) was used to generate propensity for treatment with chemo+anti-EGFR and stabilized inverse probability of treatment weights (IPTW). Balance of covariates between anti-VEGF and anti-EGFR treatment groups after stabilized IPTW was assessed using standardized differences in means for continuous variables and standardized differences in proportions for each level of binary, categorical, or ordinal variables using *pbalchk* in Stata (**eFigure 1**).[2] Standardized differences ≥ -0.10 and ≤ 0.10 were considered balanced and unlikely to confound the relationship between the exposure and outcome.

### 1.4.3. Multivariable Cox Proportional Hazards Model

This multivariable model was performed in each of the 25 imputed data sets with the treatment effect estimates combined to obtain the overall treatment effect estimate using Rubin's rules.[3] The proportional hazards assumption was tested by running the model after inclusion of an interaction term of the exposure/treatment variable with time using *tvc* in Stata. The time-exposure interaction variable was not statistically significant, and it was concluded that there was no violation of the proportional hazards assumption.

### 1.4.4. Predicted Survival Functions with Estimated Median Survival and 95% Confidence Intervals

To allow for visualization of predicted survival functions that would also provide estimations for median survival along with estimated 95% confidence intervals, bootstrapped survival functions with pointwise

© 2025 Swami N et al. *JAMA Network Open*.

95% confidence intervals at each failure time along the survival curve were generated using *bsurvci* in Stata with 1000 resampling replications.[4] Predicted survival functions were generated independently for each of the 25 imputations and subsequently averaged at each failure time per Rubin's rules.[3]

As the inclusion criteria variables RAS/RAF and tumor sidedness were imputed, each multiply imputed data set included slightly different patient cohorts. Consequently, there was variability in failure time points across the imputed data sets. When predicted survival functions over 25 imputed data sets were averaged, there were areas in the predicted survival function that appeared to "increase" due to expected variations between the imputed data sets. Due to this artifact of averaging, we specified that if the predicted survival at any time point was greater than the preceding time point, the predicted survival would be adjusted to that of the preceding time point to remove this artifact. Similarly, as the plot represents averages of the predicted survival curves rather than observed data, number at risk at each given time point are not provided. Median predicted survival in months with 95% confidence intervals was assessed by the bootstrapped point estimates averaged over 25 imputations.

## 2. eTables:

### eTable. ICD-9 and ICD-10 diagnostic codes used to define tumor-sidedness.

| ICD9 | Text | ICD10 | Text |
|---|---|---|---|
| Right-sided Primary Tumor | | | |
| 153.0 | Malignant Neoplasm of Colon; Hepatic flexure | C18.3 | Malignant Neoplasm of Hepatic flexure |
| 153.1 | Malignant Neoplasm of Colon; Transverse colon | C18.4 | Malignant Neoplasm Transverse colon |
| 153.4 | Malignant Neoplasm of Colon; Cecum | C18.0 | Malignant Neoplasm of Cecum |
| 153.6 | Malignant Neoplasm of Colon; Ascending colon | C18.2 | Malignant Neoplasm of Ascending colon |
| Left-sided Primary Tumor | | | |
| 153.2 | Malignant Neoplasm of Colon; Descending colon | C18.6 | Malignant Neoplasm of Descending colon |
| 153.3 | Malignant Neoplasm of Colon; Sigmoid colon | C18.7 | Malignant Neoplasm of Sigmoid colon |
| 153.7 | Malignant Neoplasm of Colon; Splenic flexure | C18.5 | Malignant Neoplasm of the Splenic flexure |
| 154.0 | Malignant Neoplasm of Rectum, Rectosigmoid Junction, and Anus; Rectosigmoid junction | C19 | Malignant Neoplasm of Rectosigmoid junction |
| 154.1 | Malignant Neoplastm of Rectum, Rectosigmoid junction, and Anus; Rectum | C20 | Malignant Neoplasm of Rectum |

© 2025 Swami N et al. *JAMA Network Open*.

## 3. eFigures:

### eFigure 1: Cohort Selection of Patients with RAS/RAF Wild Type Right Sided Metastatic Colon Cancer who received second-line chemotherapy with anti-VEGF vs. chemotherapy with anti-EGFR

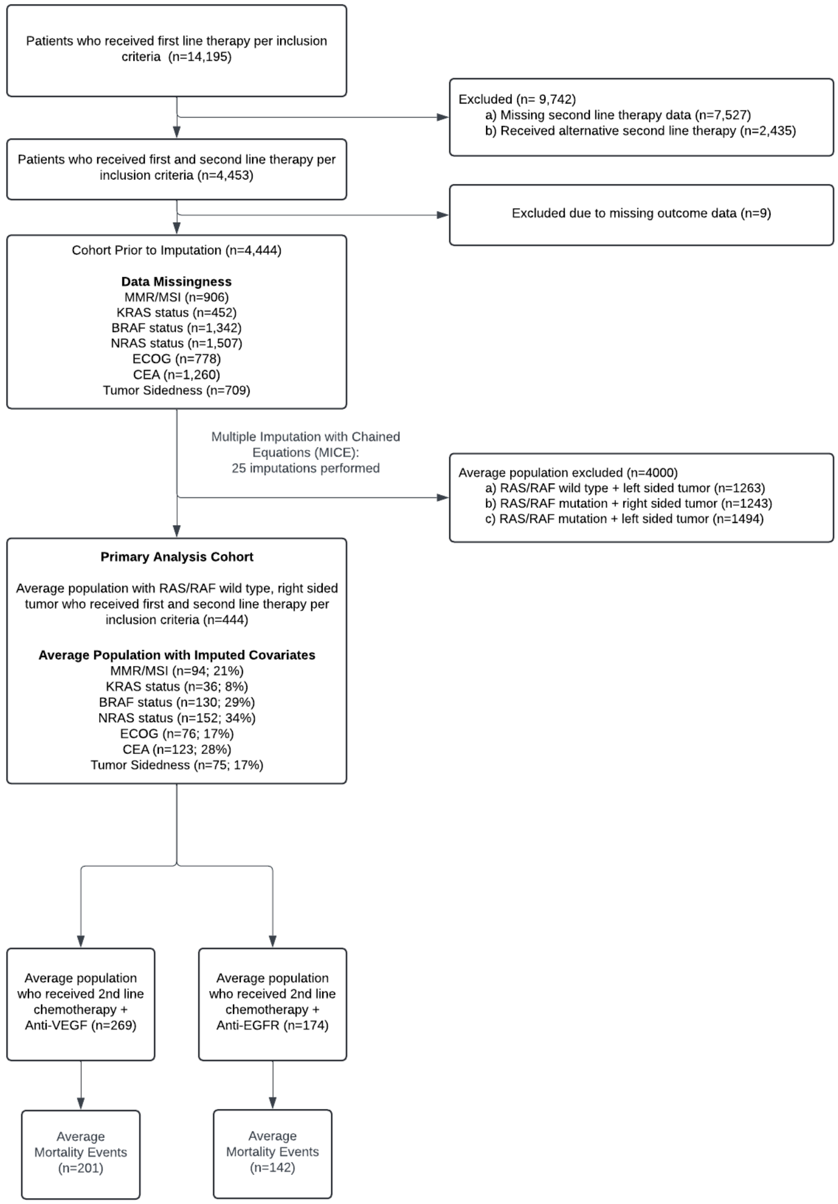


Multiple imputation with chained equations with 25 imputations was performed for RAS/RAF status, tumor sidedness, and pre-specified covariates (**eMethods 1.4.2**).

Anti-VEGF = anti-vascular endothelial growth factor – directed therapy, Anti-EGFR = anti-epidermal growth factor – directed therapy, MMR/MSI = mismatch repair/microsatellite instability, ECOG = Eastern Cooperative Oncology Group performance status, CEA = carcinoembryonic antigen

© 2025 Swami N et al. *JAMA Network Open*.

**eFigure 2: Covariate balance as assessed by standardized differences between patients receiving second-line chemotherapy with anti-VEGF and anti-EGFR therapy in the unadjusted and IPTW-adjusted analyses for the analysis cohort.**

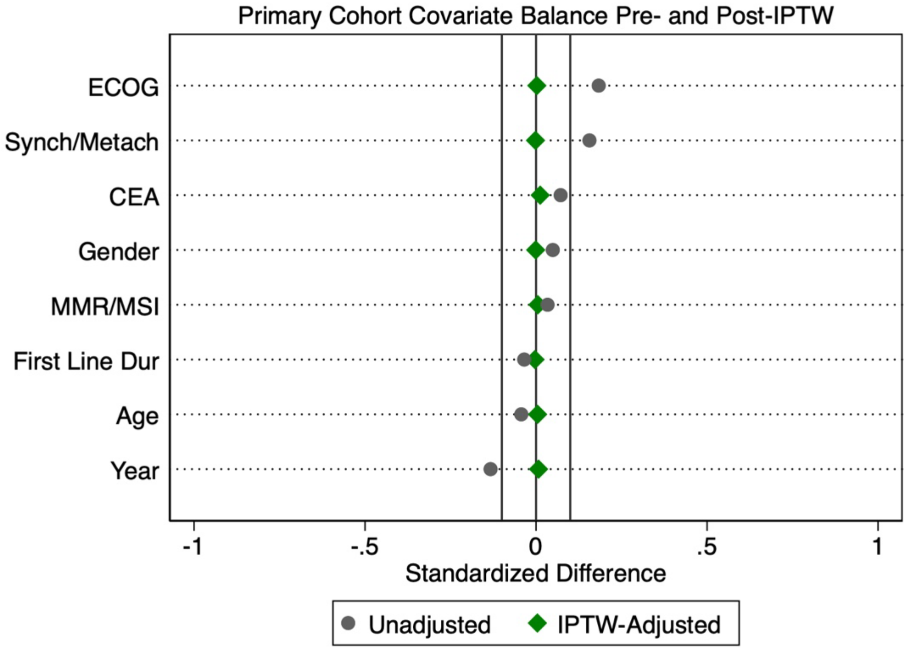


Standardized differences > -0.10 and < 0.10 are considered acceptable and unlikely to contribute to meaningful confounding. Anti-VEGF = anti-vascular endothelial growth factor – directed therapy, including bevacizumab/biosimilars; ziv-aflibercept; ramucirumab, Anti-EGFR = anti-epidermal growth factor – directed therapy, including cetuximab or panitumumab, IPTW = inverse probability of treatment weighting, ECOG = Eastern Cooperative Oncology Group performance status, Synch/Metach = Synchronous vs metachronous metastatic disease, CEA = carcinoembryonic antigen, MMR/MSI = mismatch repair/microsatellite instability, First Line Dur = duration of first line chemotherapy + anti-VEGF therapy

© 2025 Swami N et al. *JAMA Network Open*.

## 4. eReferences:


1. Luhn P, Kuk D, Carrigan G, et al. Validation of diagnosis codes to identify side of colon in an electronic health record registry. *BMC Med Res Methodol*. 2019;19(1):177. doi:10.1186/s12874-019-0824-7

2. Lunt M. Propensity Analysis in Stata Revision: 1.1:30.

3. Leyrat C, Seaman SR, White IR, et al. Propensity score analysis with partially observed covariates: How should multiple imputation be used? *Stat Methods Med Res*. 2019;28(1):3-19. doi:10.1177/0962280217713032

4. Ruhe C. Bootstrap pointwise confidence intervals for covariate-adjusted survivor functions in the Cox model. *Stata J*. 2019;19(1):185-199. doi:10.1177/1536867X19830915


© 2025 Swami N et al. *JAMA Network Open*.
