# Supplementary material for: Second-Line Treatment Strategies for Right-Sided, RAS/RAF Wild-Type Colorectal Cancer
Source: JAMA Netw Open. 2025 Jun 12;8(6):e2515087. doi: 10.1001/jamanetworkopen.2025.15087 (PMC12163663; doi:10.1001/jamanetworkopen.2025.15087)
Supplement: Supplement 2. — Data Sharing Statement [file jamanetwopen-e2515087-s002.pdf]

## Data Sharing Statement

Swami. Second-Line Treatment Strategies for Right-Sided, RAS/RAF Wild-Type Colorectal Cancer. *JAMA Netw Open*. Published online June 12, 2025. doi:10.1001/jamanetworkopen.2025.15087

### Data

**Data available:** Yes
**Data types:** Other (please specify)
**Additional Information:** Statistical Analysis and Aggregated Data regarding the cohort will be made available under discretion from Flatiron
**How to access data:** Data Requests should be made to corresponding authors and Flatiron, who will determine appropriateness of data requests and will share accordingly
**When available:** With publication

### Supporting Documents

**Document types:** Statistical/analytic code
**How to access documents:** Can be made available on direct request to corresponding author **When available:** With publication

### Additional Information

**Who can access the data:** researchers whose proposed use of the data has been approved
**Types of analyses:** For purposes of validating study findings
**Mechanisms of data availability:** With investigator support and approval per Flatiron
